# Global research trends in obesity-related asthma (2004–2023): a bibliometric analysis

**DOI:** 10.3389/fnut.2025.1528366

**Published:** 2025-04-03

**Authors:** Liye Lang, Mingxuan Ma, Hui Zhao, Jialin Zhang, Sheng Liu, Hua Liu

**Affiliations:** Department of Respiratory and Critical Care Medicine, Affiliated Hospital of Nantong University, Medical School of Nantong University, Nantong, China

**Keywords:** obesity, asthma, bibliometrics, visualization, hotspots

## Abstract

**Background:**

In recent years, an increasing body of evidence has revealed a complex interplay between obesity and asthma, prompting academic and medical communities to intensify their focus on this area of research. The objective of this study is to undertake a comprehensive bibliometric analysis of the research literature pertaining to obesity-related asthma from 2004 to 2023. This analysis aims to provide precise and valuable insights, as well as to systematically reflect upon the current status and emerging trends within the field.

**Methods:**

Literature data on obesity and asthma research was sourced from the Web of Science Core Collection database. CiteSpace and VOSviewer were utilized to visually analyze bibliometric indicators such as co-authorship, citation networks, and publication frequency of the data to facilitate the identification of patterns and trends.

**Results:**

A total of 3,118 papers were included in the analysis, encompassing 2,539 articles and 579 reviews. Throughout the last 20 years, the volume of publications has shown a consistent upward trend. The United States and Harvard University are at the forefront of this research field. Professor Dixon Anne E. is recognized as a pioneer and leading figure in the cultivation of obesity-related asthma research. Keyword analysis identified topics such as “childhood asthma,” “bariatric surgery,” “physical activity,” “gut microbiota,” “COVID-19,” “food allergy,” “asthma control,” “nutrition examination,” and “severe asthma.”

**Conclusion:**

The research domain of obesity-related asthma has experienced a substantial growth, with the United States, the United Kingdom, and China leading the global landscape. The focus on asthma in obese adolescents and children, the role of bariatric surgery, and lifestyle interventions remains a consistent area of interest, with considerable potential for further study. These findings provide a scientific basis for the development of personalized treatment programs for obese asthma patients. In addition, this study highlights the importance of further research in the fields of gut microbiota, COVID-19, and food allergy, providing directions for future policymaking.

## 1 Introduction

Asthma is a chronic inflammatory disorder of the airways, characterized by hyperresponsiveness and variable airflow limitation. Its clinical manifestations are typically recurrent wheezing, chest tightness, or cough (1). Asthma exhibits significant heterogeneity, with considerable differences in genetic predisposition, environmental exposure, clinical symptoms, pathophysiological mechanisms, and treatment responses (2). The global prevalence of asthma has been on the rise annually (3), with the current patient population estimated at approximately 358 million, resulting in approximately 500,000 deaths annually due to asthma (4, 5). Asthma not only significantly impacts patients’ daily lives and work but also imposes a substantial burden on the social economy (6, 7). Despite extensive research over the years, the etiology and pathogenesis of asthma remain complex and not fully understood (8). Recent advancements in molecular biology and immunological techniques have deepened our understanding of asthma; however, numerous unresolved mysteries require further investigation (9).

The World Health Organization defines obesity as a chronic condition with a body mass index (BMI) of more than 30 kg/m^2^ (10). Obesity is commonly attributed to excessive energy intake and expenditure, leading to the accumulation of excess body fat and an increased risk of various chronic diseases, including metabolic disorders, cardiovascular and cerebrovascular diseases, respiratory diseases, and certain cancers (11). An increasing number of articles have revealed a complex relationship with obesity-related asthma (12). Obesity may influence asthma through various mechanisms, including adipose tissue inflammation, immune system alterations, and increased respiratory system mechanical load (13, 14). Studies have shown that airway eosinophilia increases with excessive weight (15), and adipocyte-secreted factors play a crucial role in modulating airway epithelial inflammation (16). An *in vivo* study revealed that obesity affects the immune response of peripheral lymphoid organs in a murine asthma model (17). Obese asthma patients have been found to have worse lung function and a higher prevalence of wheezing symptoms compared to non-obese asthma patients (18). Together with the rising prevalence of obesity and asthma, there is an urgent need for a systematic review of research trends to fully understand the complex interactions between these conditions. This is crucial for the development of effective prevention and treatment strategies.

Bibliometrics is a comprehensive research method (19) that employs mathematical and statistical techniques to quantitatively review and analyze research within specific fields over a defined period. Bibliometric analysis enables scholars to swiftly assess the distribution of countries/regions, authors, and journals, and thereby provide a foundation for the direction and development of future research (20). Utilizing bibliometric software such as CiteSpace and VOSviewer to generate a scientific knowledge map allows new researchers to easily gain an overview of a field’s evolution and frontiers (4). Therefore, compared with other research methods, bibliometrics provides a more in-depth and comprehensive understanding in revealing the research trends and knowledge structure of the relationship between obesity and asthma. This paper aims to employ bibliometric methods to systematically sort and analyze published literature on obesity-related asthma. We hope to provide researchers with valuable insights by constructing a scientific knowledge map of obesity and asthma-related research, aiding them in understanding the current research status and frontiers, and facilitating the conduct of future research.

## 2 Methods

### 2.1 Data source and retrieval strategy

This study’s data source was the Web of Science Core Collection (WOSCC) database, which was searched on August 3, 2024. The search strategy employed the formula TS = (asthma* OR wheez*) AND TS = (overweight OR obes*), with a publication date range of January 1, 2004, to December 31, 2023.

### 2.2 Literature screening and data extraction

This literature search mainly relied on the WOSCC, in view of its status as an authoritative database of academic literature. We acknowledge that other databases such as Scopus and PubMed are equally important, but in order to maintain the consistency and pertinence of the study, we decided to use WOSCC only in this study. Future research may consider combining multiple databases to expand the scope of literature retrieval.

**Figure 1:**
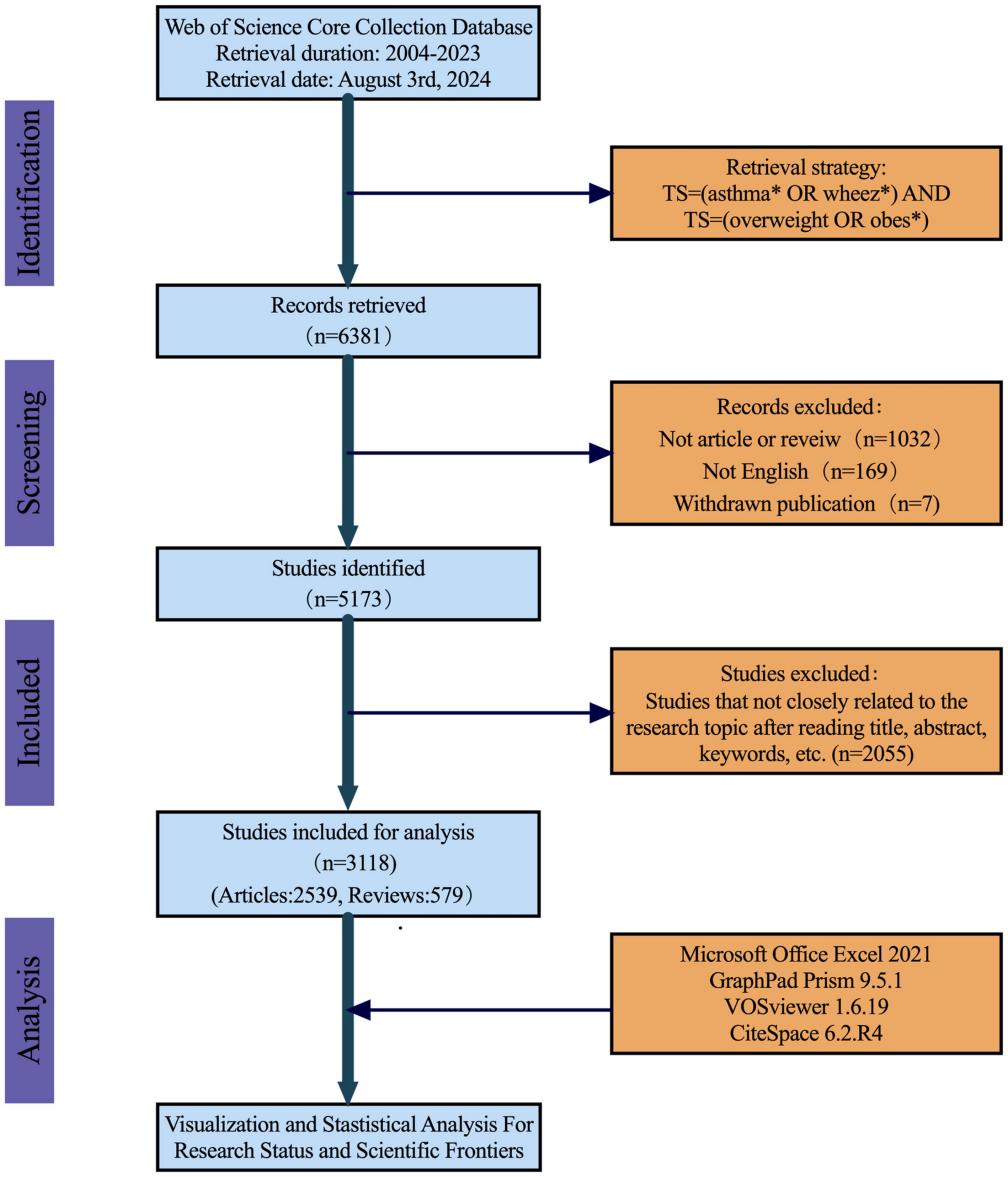
Flowchart of screening process.

Following an initial search of 6,381 records, a rigorous literature screening process was conducted. Initially, the literature type was limited to Articles and Reviews, and English was selected as the sole language. At the same time, seven retracted publications were excluded and then we got a refined dataset of 5,173 articles. Subsequently, a further screening process was employed to meticulously review the titles, abstracts, and keywords of each article to ensure compliance with the specific research topic of ‘obesity-related asthma’. Ultimately, 3,118 articles were included in this study, consisting of 2,539 original articles and 579 reviews. To mitigate the impact of daily updates, all data were downloaded and saved in “plain text file” format on the same day. The specific retrieval and screening process is illustrated in Figure 1.

### 2.3 Data analysis

Bibliometric software was used to visually analyze various indicators, including the total number of publications, publication years, countries/regions, institutions, journals, cited literature keywords, and other relevant metrics. Specifically, we used Microsoft Office Excel 2021 to summarize the publications and citations of bibliometric analysis, and made a geographical distribution map of the number of publications (21). CiteSpace (version 6.2.R4) was employed to analyze keyword bursts and citation bursts, identify emerging trends, and construct a two-map overlay of journals to assess the distribution of academic journal (22). VOSviewer (version 1.6.19) was used to analyze and draw cooperative network maps for indicators such as countries/regions and institutional collaborations (23). Data were analyzed annually from 2004 to 2023. Node selection criteria and pruning algorithm parameters were determined based on the number, frequency, and intensity of interactions. Additionally, data processing and image creation were performed using Microsoft Office Excel 2021 and Prism (version 9.5.1).

## 3 Results

### 3.1 Annual publication trends

**Figure 2:**
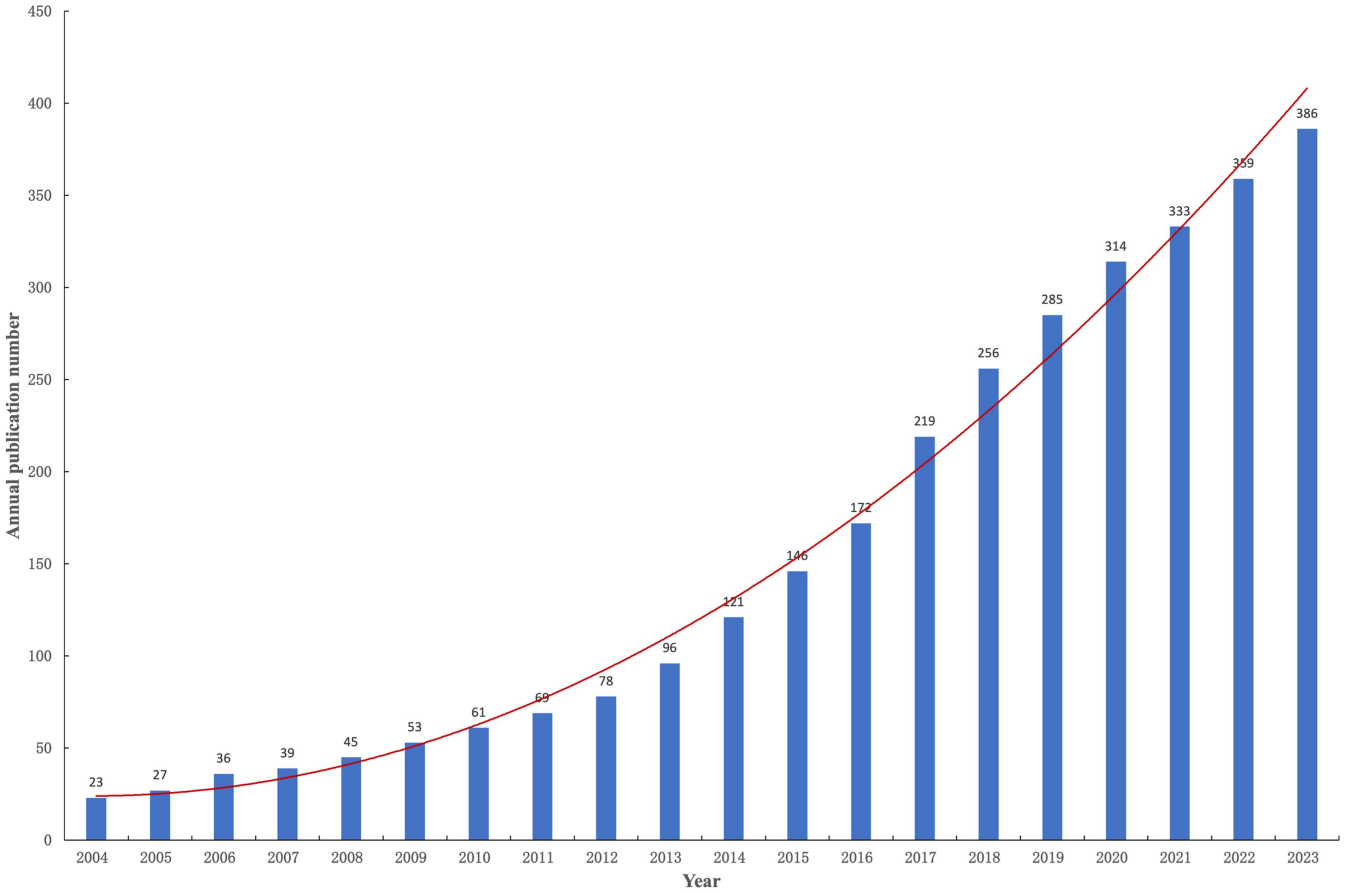
Annual publications on obesity-related asthma by years.

The annual publication trend of obesity-related asthma research not only reflects the evolution and advancement in the field but also serves as an indicator of the academic community’s focus on this area. Figure 2 depicts the trajectory of changes in the number of publications and demonstrates a consistent upward trend in the number of annual publications since 2004. In 2014, the annual number of publications first exceeded 100, and since then, it has increased by more than 15 each year. It is particularly noteworthy that from 2014 to 2023, the number of articles on obesity and asthma accounted for 83.1% of the total publications over the past two decades. Polynomial regression analysis suggests that the number of publications in 2024 is anticipated to exceed 400. These findings suggest that research on obesity and asthma has maintained its relevance and has seen a substantial increase in scholarly interest and influence since 2014.

### 3.2 Analysis of country/region contributions

The global research landscape on asthma and obesity encompasses studies from 112 countries and regions, with a total of 3,118 publications contributing to this body of knowledge. Table 1 shows a detailed breakdown of the top 10 countries/regions, which collectively account for an impressive 91.08% of all publications. This highlights these countries play a pivotal role in this field. Leading the way is the United States, which has the highest number of publications, with 1,230 articles (43.31%), followed closely by the United Kingdom with 297 articles (10.46%) and China with 244 articles (8.59%). The United States not only leads in the volume of publications but also in the number of citations, underscoring its leading position in the research hierarchy within this domain. A noteworthy observation is Canada, which, despite ranking fifth in the number of publications, has an average citation rate of 70.12, the highest among all countries. This indicates a high academic standing for the research emanating from Canadian institutions.

**Table 1:** The top 10 countries.

Country/Region	Articles	Citations	Average citation
United States	1,230	51,539	41.90
United Kingdom	297	13,342	44.92
China	244	4,926	20.19
Australia	205	8,164	39.82
Canada	166	11,640	70.12
Italy	164	4,934	30.09
Spain	141	5,682	40.30
Korea	138	2,676	19.39
Brazil	134	3,028	22.60
Sweden	121	3,392	28.03

**Figure 3:**
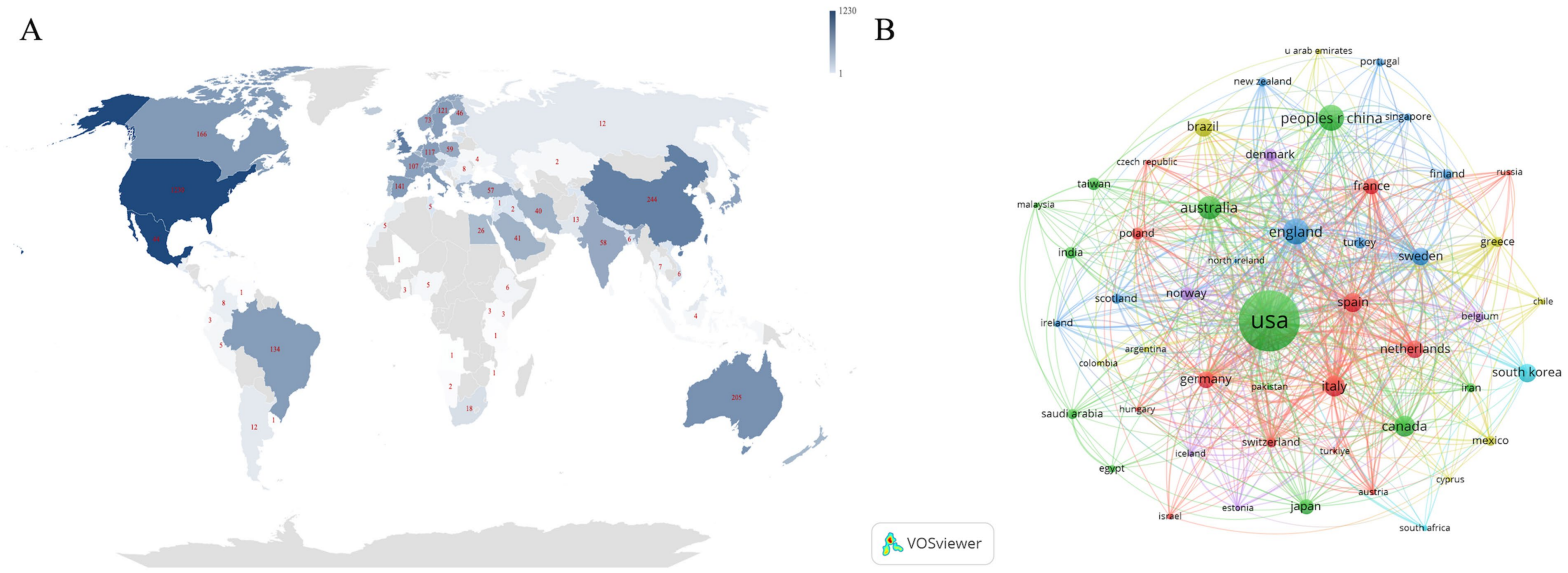
**(A)** Geographical distribution of studies on obesity-related asthma. **(B)** A visual map of cooperation among the top 50 countries/regions by publication count. Each node in the diagram symbolizes a country/region, with its size reflecting the number of articles. The thickness of lines indicates the strength of collaboration between countries.

The geographical distribution of research, as depicted in Figure 3A, reveals a concentration of significant publications in European and North American countries, such as the United States, the United Kingdom, and Canada. In contrast, Asia is represented by East Asian countries like China, South Korea, and Japan, which also contribute a substantial number of publications. Oceania and the Middle East are also represented, albeit with fewer publications. Regions such as Russia, Africa, and Central Asia have a notably lower number of articles. To further elucidate the collaborative dynamics among countries, we have constructed a cooperation network diagram for the top 50 countries/regions (Figure 3B). The diagram reveals that the United States is at the center of these collaborations, with the United Kingdom following closely. Countries closely aligned with the United States in terms of collaboration include China, Australia, the United Kingdom, and Canada. The diagram also underscores the particularly strong collaborations between European and North American countries, suggesting a dominance in scientific research within this field.

### 3.3 Productive authors and institutions

In the study of obesity-related asthma, a cumulative total of 10,628 authors have contributed to the research landscape. However, a notable trend emerges where only 14.3% of these authors have published more than one article. Among the most productive authors (Table 2), Dixon, Anne E., Camargo, Carlos A., and WOOD, Lisa G. occupy the top three positions, demonstrating a high yield of significant contributions. Notably, Forno, Erick, and Celedon, Juan C. exhibit a particularly high citation rate, surpassing that of their peers. The prominence of seven authors from the United States in the dataset reinforces the notion that the U.S. is a leading force in obesity-related asthma research, with a wealth of research outcomes.

**Table 2:** The top 10 authors.

Name	Country/Region	Articles	Citation	Average citation
Dixon, Anne E.	America	47	2,399	51.04
Camargo, Carlos A.	America	42	1,131	26.93
WOOD, LISA G	Australia	39	1985	50.90
Gibson, Peter G.	Australia	37	1713	46.30
Holguin, Fernando	America	31	1,660	53.55
Forno, Erick	America	29	2,479	85.48
Celedon, Juan C.	America	28	1920	68.57
Janson, Christer	Sweden	27	346	12.81
Rastogi, Deepa	America	25	705	28.20
Lang, Jason E.	America	25	641	25.64

**Figure 4:**
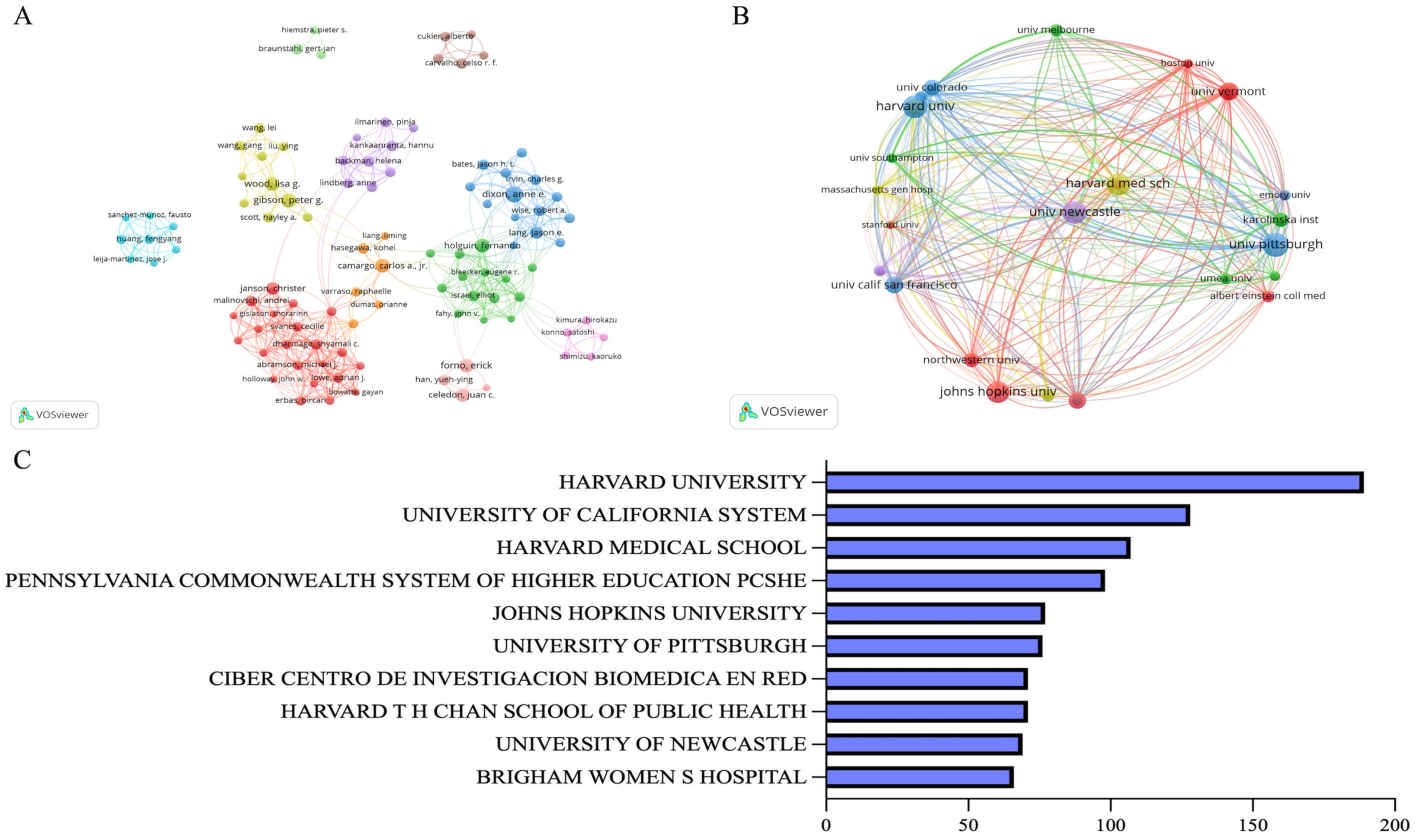
**(A)** Author cooperation network diagram. Each node represents an author. Different colors represent different author clusters. The thickness of lines reflects the level of collaboration. **(B)** Institutional cooperation network diagram. Each node symbolizes an institution, with its size reflecting the number of publications. The thickness of lines indicates the level of collaboration. **(C)** The top 10 institutions.

Figure 4A depicts the collaborative network of the top 100 authors, revealing the existence of 11 distinct author groups. While there is a strong collaborative dynamic within these groups, there appears to be a lack of inter-group collaboration and communication. This observation suggests a need for enhanced inter-agency and international cooperation among researchers in this field.

Figure 4C identifies the 10 institutions that have published the most articles in the domain. The top three institutions are Harvard University (N = 189, 19.96%), the University of California (N = 128, 13.52%), and Harvard Medical School (N = 107, 11.3%). The presence of eight university institutions in the top 10 highlights the preeminence of academic institutions in obesity-related asthma research. We utilized VOSviewer to visualize the institutional collaboration network. In order to make the cooperation network clear and more intuitive, we only shown some of the major institutions. Figure 4B clearly illustrates the communication patterns among institutions, with Harvard Medical School and Newcastle University at the core of the network, demonstrating extensive collaboration with numerous universities. Other key institutions, including Harvard University, the University of California, Johns Hopkins University, the University of Pittsburgh, and the University of Vermont, also contribute significantly to the collaborative efforts. Engaging in collaborative research with these institutions is likely to elevate the research standards and outcomes within the field.

### 3.4 Analysis of academic journals

Between 2004 and 2023, the field of obesity-related asthma has seen articles published across 864 different journals. For a more detailed examination, we have identified and analyzed the top 10 journals, with the specific data presented in Table 3. The leading journals in this field are the “Journal of Asthma,” “Journal of Allergy and Clinical Immunology,” and “Journal of Allergy and Clinical Immunology in Practice.” Notably, the “American Journal of Respiratory and Critical Care Medicine” and “Journal of Allergy and Clinical Immunology” stand out for their high total and average citation counts, suggesting that the academic contributions of these journals hold significant reference value. Seven out of the 10 top journals are classified in the JCR Q1 category, emphasizing the importance of focusing on articles published in these journals to advance research in obesity-related asthma.

**Table 3:** The top 10 journals.

Source	Articles	Citations	Average citation	IF (2023)	JCR (2023)
Journal of Asthma	172	2,996	17.42	1.7	Q3
Journal of Allergy and Clinical Immunology	83	8,447	101.77	11.4	Q1
Journal of Allergy and Clinical Immunology in Practice	73	2,405	32.95	8.2	Q1
Annals of Allergy Asthma Immunology	63	1,494	23.71	5.3	Q1
Respiratory Medicine	62	1,594	25.71	3.5	Q2
European Respiratory Journal	61	4,117	67.49	17	Q1
PloS One	60	1,587	26.45	2.9	Q1
Pediatric Pulmonology	56	902	16.11	2.7	Q2
Allergy	48	2,684	55.92	12.6	Q1
American Journal of Respiratory and Critical Care Medicine	42	5,296	126.10	19.3	Q1

**Figure 5:**
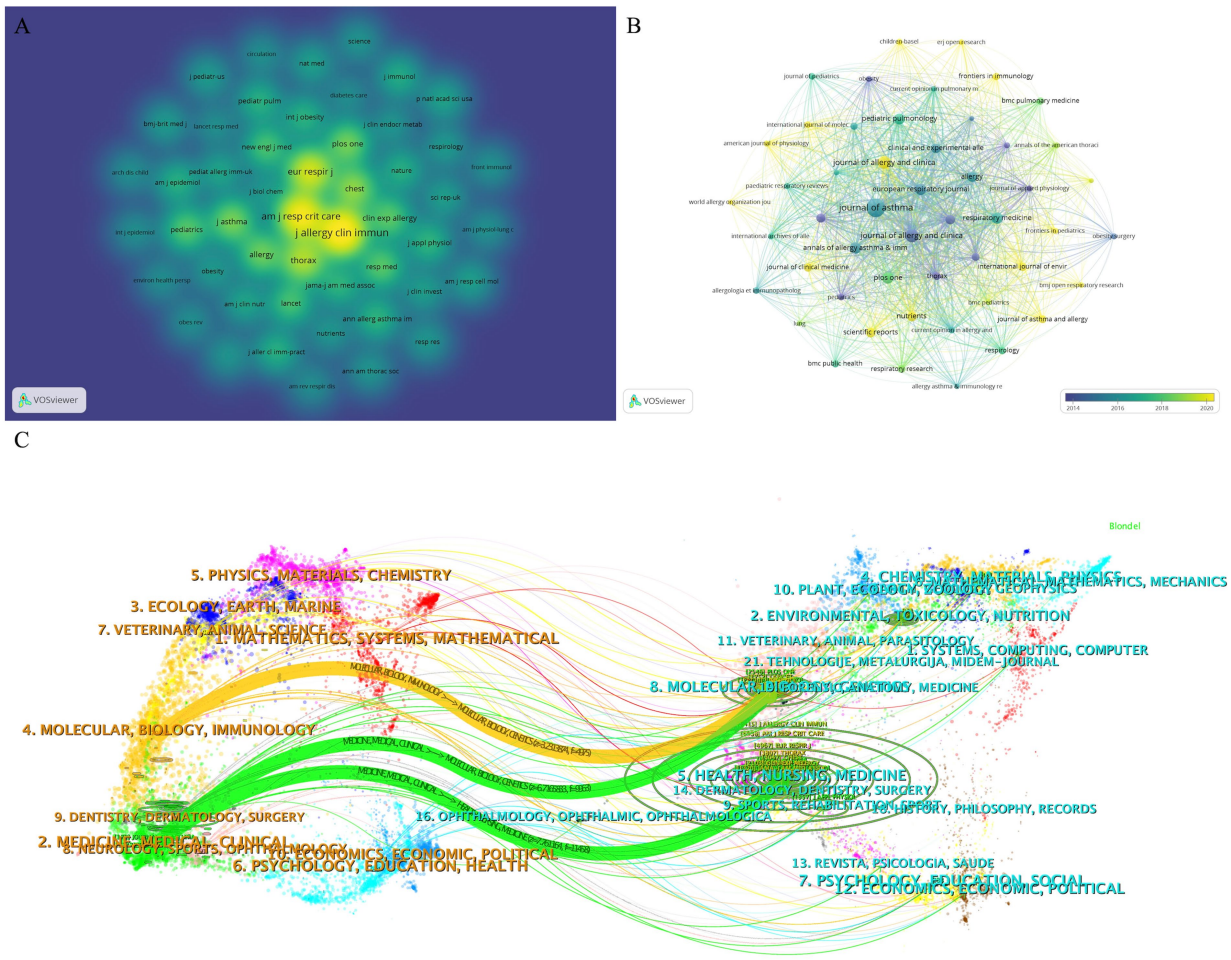
**(A)** The density distribution map of cited journal. The darker the color, the higher the density, indicating more citations. **(B)** Overlap diagram of journal citations. A node represents a journal, and the size of the node represents the amount of citation. Color represents the time change of journal citation. **(C)** The dual-map overlay of journals.

Figure 5A illustrates the density distribution of co-cited journals, with “European Journal of Respiratory Medicine,” “American Journal of Respiratory and Critical Care Medicine,” and “Journal of Allergy and Clinical Immunology” being the most frequently cited. Figure 5B presents the superposition of journal citations, highlighting journals that have seen increased citations in recent years. By paying attention to the articles in these journals, researchers can stay abreast of the latest trends and developments in the field. The double overlay diagram of journals (Figure 5C) visually represents the citation relationships, with color paths indicating the reference trajectory and knowledge flow (24). This analysis reveals that the most cited papers are predominantly from the fields of molecular biology, immunology, and clinical medicine, with a significant proportion originating from medical journals specializing in molecular biology, genetics, and healthcare.

### 3.5 Keyword and trend topic analysis

**Figure 6:**
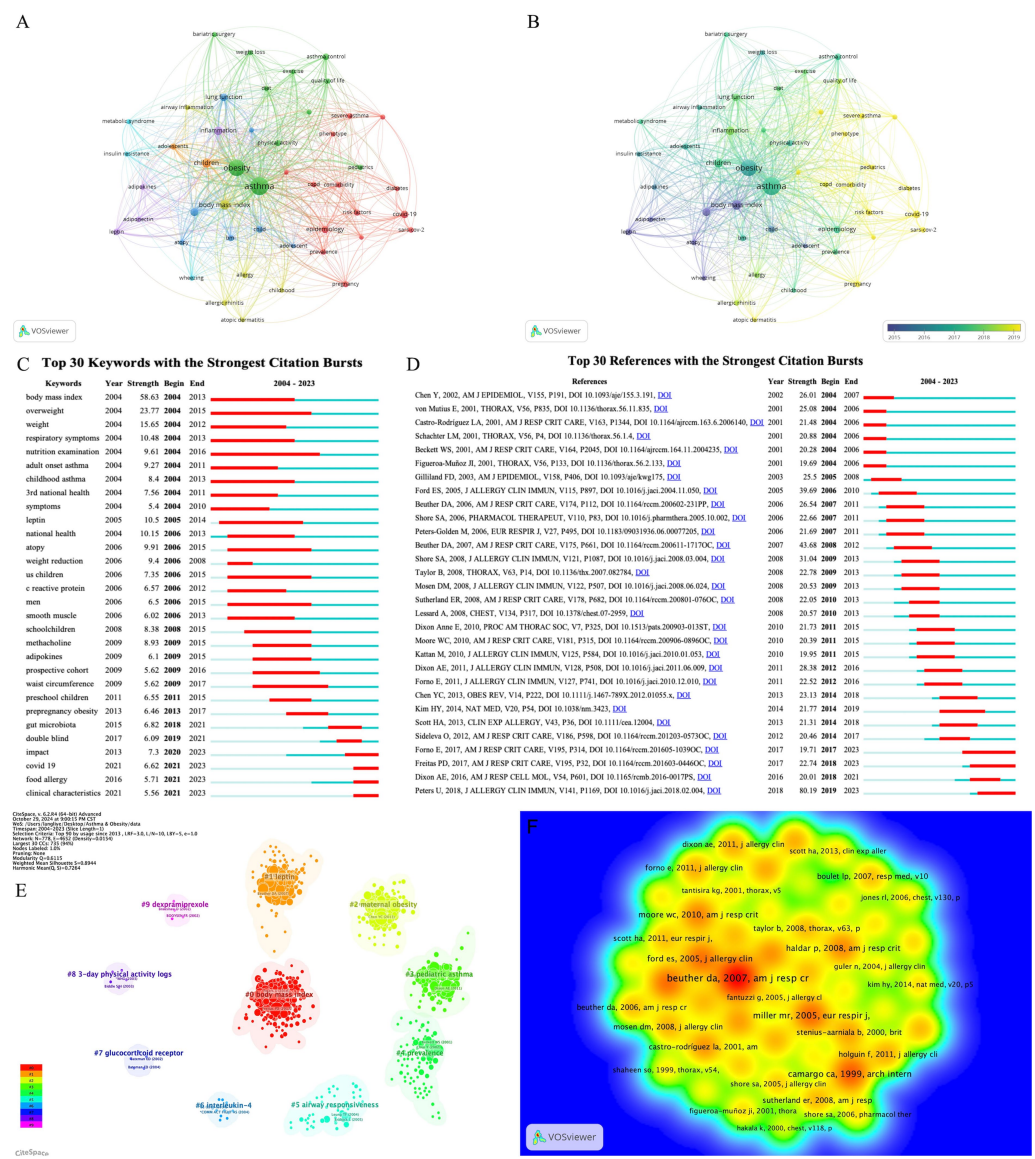
**(A)** Keyword co-occurrence network. Each node signifies a keyword, with its size indicating the frequency of occurrence. Connections between nodes represent the co-occurrence of two keywords. **(B)** Network overlap of co-occurrence keywords. Different colors represent time changes. **(C)** The map of top 30 keywords with the strongest citations bursts. **(D)** Top 30 references with the strongest citation bursts. **(E)** Keyword clustering of cited articles. **(F)** The density distribution map of article citations. The darker the color, the greater the density, indicating more citations.

Our analysis revealed a total of 328 keywords that have co-occurred more than five times within the research corpus. To visualize these interactions, we have selected the top 50 keywords and presented their co-occurrence network in Figure 6A. This network underscores the centrality of several key terms in the field, including “asthma,” “obesity,” “children,” “adolescents,” “body mass index,” “metabolic syndrome,” “bariatric surgery,” “physical activity,” “asthma control,” and “insulin resistance.” Figure 6B illustrates the evolution of keywords over recent years, highlighting terms such as ‘new coronary,’ ‘severe asthma,’ ‘pediatrics,’ ‘quality of life,’ and ‘phenotype.’ These keywords reflect the dynamic nature of the research landscape and the shifting focus of investigators within the field.

Keyword burst analysis is a powerful tool for pinpointing and tracking hot topics and emerging trends within specific research domains. It offers researchers valuable insights and academic guidance (25). Figure 6C highlights the top 30 keywords with the most significant citation bursts each lasting at least 2 years. And Table 4 summarized these keywords to observe their time evolution. The keywords ‘body mass index’ (2004–2013) ‘respiratory symptoms’ (2004–2013) and ‘nutrition examination’ (2004–2016) have garnered sustained attention over the past period. It is rather remarkable that the recent surge in the use of keywords such as “gut microbiota” (2018–2021), “COVID-19” (2021–2023), and “food allergy” (2021–2023) indicates a heightened interest in these areas. This trend suggests that these topics may soon become focal points of research poised to drive future investigations and discoveries in the field of obesity-related asthma.

**Table 4:** Keywords summary.

Keyword	Time period
Bariatric surgery	2004–2013
Body mass index	2004–2013
COVID-19	2004–2016
Food allergy	2008–2015
Gut microbiota	2013–2017
Nutrition examination	2015–2019
Physical activity	2015–2019
Prepregnancy obesity	2018–2021
Respiratory symptoms	2021–2023
Schoolchildren	2021–2023

### 3.6 Analysis of co-cited reference

Figure 6D presents the top 30 articles with the strongest citation bursts. This analysis identifies the most frequently discussed articles within a given timeframe, providing insights into the areas of obesity-related asthma research that have garnered the most scholarly interest. The trio of most frequently cited articles include “Obesity and Asthma” (Intensity = 80.19) by Peters et al., “Obesity and Asthma” (Intensity = 43.68) by Beuther et al. and “The Epidemiology of Obesity and Asthma” (Intensity = 39.69) by Ford et al. Notably, the review by Peters et al. remains a highly relevant and influential piece, offering a comprehensive overview of the potential mechanisms underlying obesity-related asthma and providing targeted recommendations for addressing related issues. In parallel, this study employed CiteSpace to cluster the keywords of the cited literature. Figure 6E illustrates that the cited references are grouped into 10 distinct research clusters, encompassing key terms such as “body mass index,” “leptin,” “physical exercise,” and “glucocorticoid receptor.”

Figure 6F depicts the density distribution of the most frequently cited references. It becomes apparent from the visualization that articles by Beuther DA, published in “AMJ RESP CR” in 2007, Miller MR, published in “EUR RESPIR J” in 2005, and Camargo CA, published in “ARCH INTERN” in 1999, continue to hold substantial reference value despite their earlier publication dates. This underscores the enduring significance of these works within the field of obesity-related asthma research, even as new findings and insights emerge.

## 4 Discussion

Over the years, asthma has significantly impacted individuals and society, making it a major global health issue (26). Previous research has consistently shown a strong link between obesity and asthma, leading to a surge in scholarly interest in the interplay between these two conditions. In this study, we developed an innovative interdisciplinary network analysis framework, which integrates bibliometric indicators to elucidate the intricate interconnections among various disciplines within the realm of obesity-related asthma. This approach not only monitors the temporal progression of research trends but also ensures that the derived findings are grounded in robust theoretical foundations and are seamlessly aligned with the practical applications in clinical nutrition. Through the meticulous analysis of 3,118 articles meticulously selected from the WOSCC, utilizing bibliometric tools such as CiteSpace and VOSviewer, we gained a comprehensive insight into the trajectory of obesity-related asthma research. This analysis has also delineated the current research hotspots and identified potential future research avenues within the field.

### 4.1 General information

The 3,118 articles retrieved from the WOSCC database were disseminated across 864 academic journals. These articles were authored by 11,062 researchers affiliated with 4,223 institutions spanning 112 countries and regions. Our analysis revealed an increasing trend of research publications on obesity-related asthma over the past two decades. Notably, prior to 2014, the volume of related research articles was relatively limited. After that, annual publications increased significantly, which may be attributed to the concurrent rise in the prevalence of both obesity and asthma. This escalation in interest suggests that researchers are increasingly focusing on this area due to the growing public health implications of these conditions.

The distribution of scholarly publications across different countries serves as a proxy for assessing the academic prowess within a given field. Our analysis indicates that the United States holds a commanding lead in obesity-related asthma research, with 39.45% of the publications in this study originating from the it. The U.K. and China follow closely, each contributing a substantial number of research articles to the field. This may be due to their high incidence, strong economic support, high-quality education, favorable policies and international cooperation. The U.S.’s central role in the international research collaboration network within this domain underscores its status as a core hub for international exchange, which may be closely related to its advanced research infrastructure and a large amount of research funding. It is particularly noteworthy that while China ranks among the top three in the number of articles, the average citation rate of its publications is notably lower than that of other countries. This phenomenon may be due to the lack of targeted academic funds in China, so that the research depth and influence of the paper are limited. This suggests a need for China to enhance the quality of its research outputs. The disparity in research levels between developed countries like the U.S. and developing countries such as China may be linked to the level of governmental attention and financial support allocated to this area (27).

Among the top 10 authors, seven contributors come from the United States. Professor Dixon, Anne E. has been instrumental in advancing the study of obesity-related asthma and is considered a pioneer and a leading figure in the field. Professor Forno, Erick not only leads in the total citations of articles but also boasts a significantly higher average citation rate, reflecting his substantial influence in the field. The high citation rates of the top 10 authors’ works underscore their outstanding contributions to the field. The research trends of these authors can provide valuable insights into the key issues and in obesity-related asthma research. Tracking the research trends of these authors will help to grasp the core issues and cutting-edge developments in obesity-related asthma research. Our study also revealed that in the co-author network clustering map, researchers predominantly collaborate within their respective clusters, with limited inter-cluster cooperation and communication. The obstacles that cause this dilemma of cooperation and communication may include cultural differences, uneven distribution of resources, and intellectual property rights. In order to promote effective cross-agency cooperation, it is recommended to establish cross-agency cooperation committees to coordinate resources and promote communication. And formulate clear intellectual property sharing agreements to solve the problem of resource allocation and intellectual property protection. Institution analysis shows that the U.S. boasts eight institutions in the top 10, further testament to its exceptional scientific research capabilities in this area. Harvard University, a world-renowned academic institution, has published the most articles in this field. Within the network of these leading institutions, Harvard Medical School and the University of Newcastle serve as key nodes for collaboration and exchange, significantly contributing to the field’s development and breakthroughs.

The consumption of high-quality, authoritative journal articles is a cornerstone for researchers seeking to stay at the forefront of their field. Such articles provide access to the latest research outcomes, cutting-edge technological advancements, and theoretical advancements, which are essential for enhancing one’s research proficiency and fostering the growth of related disciplines. In the context of obesity-related asthma research, the selection of appropriate journal articles is of paramount importance. Table 3 indicates that four of these top 10 journals boasting impact factors exceeding 10. Notably, the “Allergy and Clinical Immunology,” the “European Respiratory Medicine Review,” and the “American Journal of Respiratory and Critical Care” are highly regarded journals in the field, renowned for their quality and authority. Focusing on and engaging with the high-quality articles published in these journals will undoubtedly be of significant benefit to scholars engaged in obesity-related asthma research (28).

The journal knowledge flow diagram depicted in Figure 5C illustrates that the journals contributing to this field are predominantly found within medical disciplines such as molecular biology, immunology, clinical medicine, and healthcare. When this information is synthesized with the data presented in Table 3, it becomes evident that allergy, immunity, and clinical medicine represent the primary research avenues within obesity-related asthma. This underscores the interdisciplinary nature of the field and the importance of these core areas in driving forward the scientific understanding and treatment of this complex condition.

### 4.2 The evolution of research on obesity-related asthma

An in-depth analysis of the cited literature reveals the progressive development of research on obesity-related asthma. Early research established a compelling association between obesity and asthma. For instance, a study from 2004 suggested that an increase in BMI could influence the pathogenesis of asthma by altering the mechanical properties and inflammatory mechanisms of the respiratory system—a process that is closely linked to obesity (29). An epidemiological study indicated that obese children are more likely to develop asthma than non-obese children (30). A prospective meta-analysis by Beuther et al. (31) revealed a dose–response relationship between overweight and obesity and the incidence of asthma in both genders, suggesting that interventions aimed at reducing obesity could significantly decrease asthma prevalence.

In 2010, Dxion et al. (32) pointed out that obesity has an important impact on lung function parameters. A clinical prospective study by Dxion et al. (33) in 2011 showed that bariatric surgery can significantly improve the symptoms of obese asthma patients, and it is necessary to develop specific treatment strategies for such patients. Since then, clinical controlled studies have increasingly delved into the role of weight loss in asthma management (34, 35). Peters et al. (26) emphasized the mechanism research focusing on diet, gut microbiota, genetics and epigenetics, as well as the treatment research exploring weight loss surgery and lifestyle intervention.

Figure 6E categorizes the citations into research clusters such as “body mass index,” “leptin,” “physical exercise,” “airway hyperresponsiveness,” “epidemiology,” and “glucocorticoid receptor.” These clusters correspond to the epidemiological, lifestyle intervention, and treatment studies discussed previously, collectively depicting the trajectory and key research areas within the field of obesity-related asthma.

### 4.3 Hotspots and future trends

The visual analysis of keywords within the article has illuminated the current hotspots and emerging trends in the field of obesity-related asthma. The network of top 50 co-occurrence keywords prominently features terms such as “asthma,” “obesity,” “children,” “adolescents,” “body mass index,” “metabolic syndrome,” “bariatric surgery,” “physical activity,” and “asthma control.” These keywords highlight the focal points of research, including the impact of obesity on childhood asthma, the management of asthma control, the role of bariatric surgery, and lifestyle interventions. Notably, the outbreak of COVID-19 in recent years has introduced “COVID-19” as a new keyword in the research landscape. The influence of COVID-19 on obese asthma patients and the underlying mechanisms are areas that require further investigation to uncover unique health risks and treatment requirements. Next, we will delve deeper into a comprehensive analysis and discussion of these trending topics.

The increasing prevalence of obesity in children and adolescents has garnered sustained attention, particularly in the context of asthma management. A Mendelian randomization study has identified a significant risk association between obesity and childhood asthma, emphasizing the importance of improving sleep quality and preventing obesity-related complications in children to mitigate asthma exacerbations (36). Obesity is associated with persistent asthma symptoms and distinct inflammatory profiles in pediatric patients, with traditional treatments showing limited efficacy in improving asthma control and lung function (37). Innovative therapeutic approaches to weight loss of children with obese-related asthma are crucial in prevention and control. It is noteworthy that maternal obesity prior to pregnancy is a known risk factor for asthma in the offspring (38, 39). Furthermore, an excessive weight during the early infancy period is also linked to a heightened risk of developing childhood asthma (40). Extensive research has demonstrated that the metabolic syndrome is associated with a marked decrement in lung function parameters among patients suffering from obesity-related asthma (41). The management of metabolic syndrome to alleviate asthma symptoms is thus a promising area. Moreover, the intimate connection between metabolic syndrome and obesity makes the need for a deeper exploration of its implications for obese individuals with asthma.

For those seriously overweight adult asthma patients, weight control is imperative. Studies have demonstrated that comprehensive weight loss strategies, including diet control, exercise, and psychological interventions, can significantly improve the symptoms of asthma (42). Weight loss interventions are associated with clinical improvements in asthma control, quality of life, and overall health-related quality of life (43). A clinical study has shown that serum inflammatory markers in asthma patients decrease significantly following weight loss (44). Effective weight loss measures for asthmatic patients with obesity primarily involve weight loss surgery and lifestyle interventions, which can significantly loss weight and enhance respiratory function, thereby effectively controlling asthma symptoms (45, 46). However, weight loss surgery in severely obese asthma patients necessitates careful preoperative evaluation, postoperative care, lifestyle adjustments, and regular follow-up to mitigate the risk of complications (47).

Dietary adjustments and moderate physical activity are pivotal components of lifestyle interventions for asthmatic patients with obesity. Research indicates that regular physical activity can significantly control asthma symptoms in patients with appropriate exercise regimens, compared to obese patients without exercise (48). The incidence of asthma decreased and lung function improved in those asthmatic patients with obesity after receiving physical activity interventions, suggesting the necessity of ongoing research in this field (49).

The keyword explosion analysis reveals that gut microbiota, COVID-19, and food allergy have been areas of ongoing focus over the past 2 years. The global health crisis of the COVID-19 pandemic has emphasized the importance of obesity as a risk factor for various diseases, including respiratory diseases such as asthma. In this context, the intestinal microbiota has received unprecedented attention due to its key role in regulating host immune response and inflammation and the continuous advancement of technology. Studies have shown that gut microbiota diversity and species changes are associated with obesity and asthma (50). A cohort study has demonstrated that gut microbiota can regulate the immune response to LPS in asthmatic patients with the 17q21 risk allele (51). The ecological imbalance of gut microbiota may increase the risk of asthma or aggravate asthma symptoms in obese individuals by regulating immune response and inflammation levels, and its regulation could be a key early intervention and prevention strategy for obesity-related asthma (52). Therefore, exploring the use of probiotics and prebiotics to restore healthy gut microbiota in obese asthma patients may help reduce the risk of respiratory infections and improve overall immune function. Extensive clinical and basic research is needed to explore this potential therapeutic avenue in the future.

A retrospective study has found that obese asthma patients are at a higher risk of COVID-19 infection and often experience more severe pathological manifestations (53). Another study indicates that asthmatic children with obesity exhibit a greater decline in lung function and more severe clinical symptoms following COVID-19 infection compared to general asthmatic children (54). The metabolic disorders and abnormal immune function in obese patients may contribute to increased susceptibility to COVID-19, and the condition may worsen post-infection. Asthmatic patients with obesity also face heightened respiratory sensitivity, which may exacerbate respiratory symptoms and complications upon COVID-19 infection (55). Therefore, asthmatic patients with obesity are at a higher risk of infection, necessitating special attention and the strengthening of protective measures and targeted treatments. We recommend a comprehensive lifestyle plan, including a balanced diet, regular exercise and stress control, to help obese asthma patients lose weight and improve lung function, and emphasize the importance of smoking cessation to reduce asthma exacerbations. In-depth research into the health risks and treatment needs of obese asthma patients during the pandemic will aid in preparing for future public health events.

The interplay between food allergies, obesity, and asthma is a complex area of research, with multiple mechanisms proposed to mediate their interconnected development. These include immune system overreactions and the exacerbation of inflammatory processes. Epidemiology studies have consistently demonstrated a heightened co-morbidity rate among patients suffering from both obesity and asthma, who also have food allergies, indicating a plausible interplay between these conditions (56). By investigating the precise pathways through which food allergies may influence the progression of obesity and asthma, researchers can identify novel therapeutic targets and develop interventions that may mitigate the severity of these conditions and prevent their onset in susceptible populations (57). Future research endeavors should be directed towards elucidating the association mechanisms between food allergies and obesity-related asthma, as well as towards the development of targeted preventive and therapeutic strategies.

### 4.4 Limitations

Admittedly, our research has some limitations in bibliographic analysis. Initially, our exploration was confined to the WOSCC database, and literature coverage and update speed may not be as comprehensive and timely as Scopus. This could lead to an incomplete representation of the research landscape and introduce potential biases in the identification and inclusion of studies. Furthermore, while our study aimed to encompass the majority of relevant research, due to the possible selective bias in the inclusion process of the database, the frontier research results of some emerging fields or specific disciplines had not been fully included, resulting in a slight lack of representativeness of recent research. Moreover, our bibliometrics method focused on the quantity of literature rather than quality. While this approach can provide useful insights into research trends and impacts, it may not capture all of the important research contributions, especially those that are not widely cited but have significant impacts.

## 5 Conclusion

In summary, this comprehensive review of the literature on obesity-related asthma delineates the key features, recent advancements, and pivotal concerns within the field. It offers a robust foundation for future research and decision-making by highlighting the evolution of the field over the past two decades. The United States and Harvard University stand out as a leader in this area. Professor Dixon Anne E.is recognized as a foundational figure, a pioneer, and a key developer of this discipline. Our study suggests that childhood asthma in the context of obesity, as well as the implications of weight loss surgery and life-style interventions are important research directions in this field. In addition, considering the benefits of nutritional intervention in the management of obesity and asthma, we particularly point out that this field is expected to become a key focus in future research. Current frontier topics include gut microbiota, COVID-19, and food allergies, necessitating further extensive clinical and basic research. In the face of these challenges and opportunities, we strongly recommend prioritizing interdisciplinary research cooperation and significantly increasing capital investment in emerging research fields. This will not only promote an in-depth insight into the complex interrelationship between obesity and asthma, but also lay a solid foundation for the development of innovative treatment strategies and public health interventions.

## Data Availability

The original contributions presented in the study are included in the article/supplementary material, further inquiries can be directed to the corresponding author.
